# Human immunodeficiency virus-negative plasmablastic lymphoma: A comprehensive analysis of 114 cases

**DOI:** 10.3892/or.2015.3808

**Published:** 2015-02-17

**Authors:** MIN LIU, BAILONG LIU, BIN LIU, QIANG WANG, LIJUAN DING, CHENGCHENG XIA, LIHUA DONG

**Affiliations:** 1 Department of Radiation Oncology, The First Hospital, Jilin University, Changchun, Jilin 130021, P.R. China; 2 Department of Hand Surgery, The First Hospital, Jilin University, Changchun, Jilin 130021, P.R. China

**Keywords:** clinicopathological features, plasmablastic lymphoma, HIV negative, etiology, treatment, prognosis

## Abstract

Human immunodeficiency virus-negative plasmablastic lymphoma (PBL) is an extremely rare entity. Its clinicopathological features, optimal treatment strategy and prognostic factors remain obsure. An extensive search was performed in the English language literature within the Pubmed database using the key words: ‘plasmablastic lymphoma and human immunodeficiency virus-negative or immunocompetent’. Data from 114 patients from 52 articles were analyzed. The mean patient age at diagnosis was 58.90 years (range, 2–86). HIV-negative PBL showed a predilection for elderly individuals (patients older than 60 years, 56.14%) and affected more males than females (M:F, 2.29:1). Ann Arbor stage IV patients accounted for 39.22% while bone marrow involvement was less frequent (12.79%). The Ki-67 index was high with a mean expression of 83%. Epstein-Barr virus (EBV) infection was common being positive in 58.70% of the patients while herpesvirus-8 (HHV-8) infection was rare being positive in only 7.55% of the patients. Immunosuppression was noted in 28.16% of patients. The median overall survival (OS) was 19 months. The 1- and 2-year survival rates were 52.3 and 45.3%, respectively. Age, gender and primary site showed no strong relationship with OS while Immunosuppression, Ann Arbor stage IV and EBV negativity were able to predict a poorer OS. Either complete remission (CR) or partial remission (PR) was superior to the refractory group in OS (P<0.0001 and P=0.0066, respectively). For stage I patients, the application of radiotherapy did not improve the OS. In conclusion, HIV-negative PBL is a distinct entity likely occurring in elderly and immunosuppressed individuals. Immunosuppression status, Ann Arbor stage IV, EBV negativity and refractory to treatment are poor prognostic factors of OS in HIV-negative PBL.

## Introduction

Plasmablastic lymphoma (PBL) was firstly identified as a unique clinicopathological entity by Delecluse *et al* (1). For a long time, PBL was viewed as a disease exclusively involving the oral sites of human immunodeficiency virus-positive individuals. Recently, more and more cases of PBL in immunocompetent patients have been reported. However, there is little consensus concerning many aspects such as etiology, clinical findings, optimal treatment strategy and prognostic factors of HIV-negative PBL. Furthermore, Castillo *et al* demonstrated that HIV-negative PBL cases are rather different compared with their counterparts (2). Through an extensive literature search, 114 cases of HIV-negative PBL were described. To our knowledge, this is the most comprehensive analysis concerning PBL in HIV-negative patients. Our study provides a full-scale view and helps to expand our understanding of this unique lymphoma.

## Patients and methods

### Literature review

An extensive search was carried out in Pubmed using the key words: ‘plasmablastic lymphoma and human immunodeficiency virus-negative or immunocompetent’ in the English language literature. Only cases with definite pathologic diagnosis of PBL and description of no HIV infection were enrolled. A total of 114 cases of HIV-negative PBL were described in case reports or in small sample size case analyses between February 1997 and 2014 (1,3–53).

### Data retrieval

Data were retrieved according to characteristics such as age, gender, stage, site, bone marrow involvement, Ki-67 expression, pathological findings, Epstein-Barr virus (EBV) infection, herpesvirus-8 (HHV-8) infection, immunosuppression, treatment strategy (chemotherapy, radiotherapy and surgery), treatment response, survival and prognosis. Chemotherapy included treatment with bortezomib, but excluded treatment merely with steroids. Surgery excluded incisional biopsy. Ki-67 expression with exact values was recruited, and data showing values of 1+, 2+, 3+ and 4+ were excluded. Immunohistochemistry (IHC) with ± was viewed as positive expression. Complete remission (CR) included near CR. The period from diagnosis to death or latest follow-up was considered as overall survival (OS).

### Statistical analysis

Cumulative survival was expressed by Kaplan-Meier estimates and compared with the log-rank (Mantel-Cox) test. SPSS 15.0 statistical software was used for data analysis. P-value <0.05 was indicative of a statistically significant result.

## Results

### Clinical features

HIV-negative PBL occurred in a wide spectrum of patients, aged from 2 to 86 years, with a mean age at diagnosis of 58.90 years. Notably, HIV-negative PBL mostly occurred in the elderly population. As Table I shows, patients older than 60 years accounted for 56.14% of all the cases. PBL was rarely present in young immunocompetent individuals especially children and teenagers (only 3 patients). HIV-negative PBL was more common in males with a male-to-female ratio of 2.29. With respect to clinical stage, stage IV was most common and I was secondary. Although stage IV was noted in 39.22% of the patients, bone marrow involvement was present in only 12.79% patients. As far as the primary site was concerned, the majority was extranodal. The oral cavity, nasal cavity and sinus were 2 most common sites of involvement. A total of 15.79% of the cases involved the gastrointestinal tract.

### Etiological analysis

As shown in Table II, EBV infection was common in HIV-negative PBL, involving 58.70% of the patients, while HHV-8 infection was rather rare, being positive in only 4 cases. Immunosuppression status including post-transplantation, immune-related disease and current or previous malignancy was noted in quite a number of patients (28.16%).

### Pathological findings

As shown in Table III, plasma cell markers CD38, VS38c, CD138 and MUM1 were expressed universally in PBL without HIV infection. EMA and CD45 were variably expressed, being positive in 59.26 and 40% of the cases, respectively. B-cell marker CD20 was rarely noted (only 1 case +, 4 cases ±). A total of 37.93% of the cases had CD79a expression. Notably, a small number of cases expressed T-cell markers CD3 and CD5; 14.89 and 13.04%, respectively. Furthermore, a high number of patients (21.43%) expressed NK-cell marker CD56. Ki-67 expression was universally high with a mean value of 83%, indicating an aggressive behavior.

### Treatment and prognosis

As Table IV demonstrates, 18.52% of the patients underwent surgery. A total of 34.44% of the patients received radiotherapy. A majority of the patients (84.27%) received chemotherapy. The CR, partial remission (PR) and refractory rates were 54.93, 16.90 and 25.35%, respectively.

### Prognostic factors of OS

**Figure 1:**
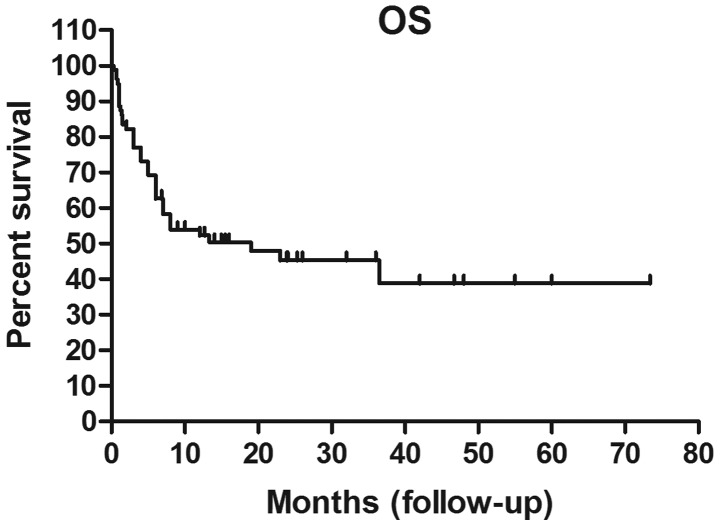
Overall survival (OS) curve (n=80).

As shown in Fig. 1, the median OS was 19 months. The 1- and 2-year survival rates were 52.3 and 45.3%, respectively.

**Figure 2:**
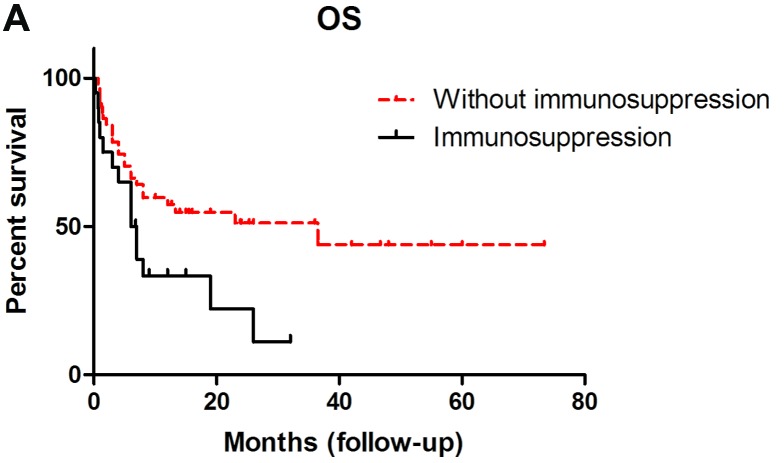

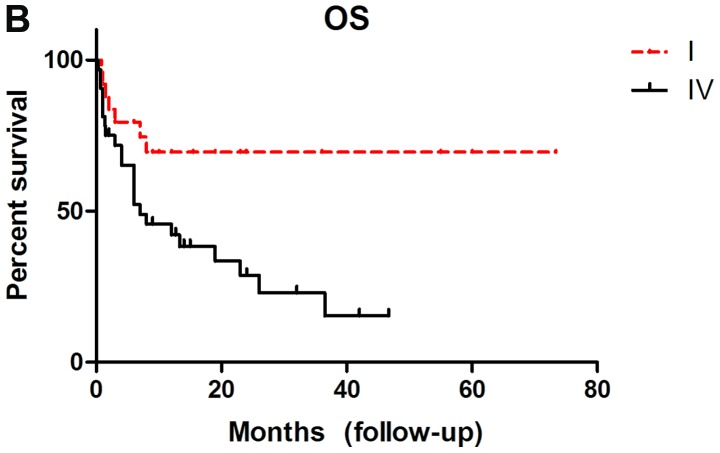

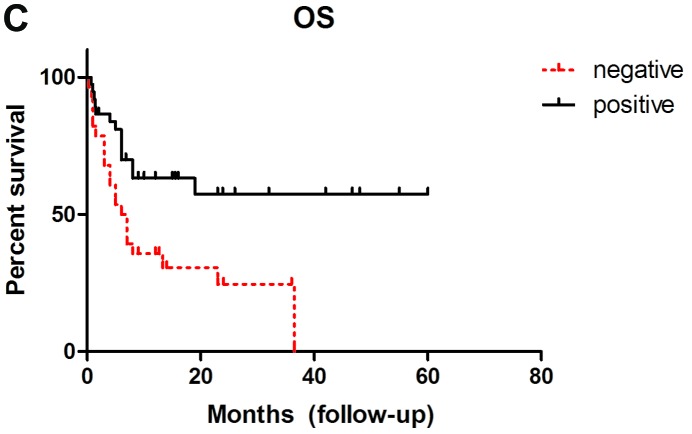
Immune status, Ann Arbor stage and EBV infection status affect overall survival (OS) (hazard ratio, 0.4114, P=0.0223; hazard ratio, 0.3731, P=0.0079; hazard ratio, 2.763, P=0.0046, respectively).

Immunosuppression was a poor prognostic factor of OS. As Fig. 2A demonstrates, patients without immunosuppression had an apparently better OS than their immunosuppression counterpart with a median survival of 36.5 vs. 6.5 months. As far as Ann Arbor stage was concerned, stage I showed an OS advantage over stage IV (P=0.0079, Fig. 2B). EBV negativity was another poor prognostic factor of OS. As Fig. 2C shows, HIV negative PBL patients without EBV infection had a much poorer OS than EBV positive patients (P=0.0046).

**Figure 3:**
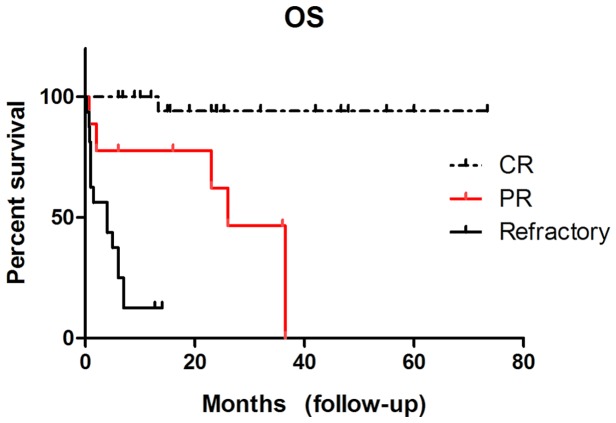
Treatment response had a strong association with overall survival (OS).

Treatment response had a strong association with OS. As shown in Fig. 3, either CR or PR was superior to the refractory group in OS (P<0.0001 and P=0.0066, respectively). Furthermore, the CR group showed an OS benefit when compared with PR (P=0.001).

Univariate analysis (Table V) revealed that age, gender and primary site had no strong relationship with OS. Ann Arbor stage IV, immunosuppression, EBV negativity and refractory to treatment were poor prognostic factors of OS. For stage I patients, the application of radiotherapy did not improve the OS.

## Discussion

In 1997, Delecluse *et al* first described 16 PBL cases (15 HIV-positive and 1 HIV-negative) (1). PBL was once considered as a malignancy that predominantly occurred in HIV-infected individuals. In 2010, Castillo *et al* analyzed 71 cases of HIV-negative PBL published before August 2009 and first revealed that HIV-negative PBL had distinct clinicopathological features (2). Soon after, Castillo *et al* (54) and Liu *et al* (26) reviewed 76 and 79 cases of HIV-negative PBL published before June 2010. In recent years, with increasing awareness of this unique entity, more cases have been reported in diverse sites in patients without HIV infection. Thus, it is necessary for us to retrieve all the cases published to date to get a full-scale understanding of this unique lymphoma.

With respect to clinicopathological features of HIV-negative PBL, Ann Arbor stage I was rather predominant, accounting for 31.37% of the entire enrollment, higher than a previous study by Castillo *et al* (2) (23%), and similar to a study by Liu *et al* (26) (32.9%). Furthermore, stage I showed a OS superiority than stage IV (P=0.0079). Primary skin HIV-negative PBL accounted for a smaller proportion, only 6.14%, when compared with a previous study of 12% (54). More patients (18.52%) received surgery. Castillo *et al* reported that only 4% of the patients underwent surgery (54). CD56 expression in our research was 21.43%, much higher than Castillo *et al* findings (6%) (2). Furthermore, a portion of HIV-negative PBL expressed T-cell markers such as CD3 (14.89%) and CD5 (13.04%).

Regarding the aspect of etiology, some studies have indicated that PBL is closely related with EBV infection (55). Our study also confirmed that 58.70% cases of HIV-negative PBL exhibited EBV infection. Furthermore, univariate analysis showed that EBV infection was a good prognostic factor of OS (P=0.0046). The concrete mechanism need to be clarified in the future. Furthermore, only 7.55% of patients were HHV-8-positive, indicating that HHV-8 infection has a negligible role in HIV-negative PBL. On the contrary, we found that immunosuppression contributed to 28.16% of the cases including post-transplantation, immune-related disease and current or previous malignancy. Additionally, immunosuppression was a poor prognostic factor for OS. Patients with immunosuppression had a poorer OS with a median survival of 6.5 months while patients without immunosuppression achieved a median survival of 36.5 months. Our data also showed that HIV-negative PBL had a predilection for elderly individuals (patients older than 60 years, 56.14%). This proportion in our research was higher than that noted in the studies of Liu *et al* (43%) (26) and Castillo *et al* (49%) (54). Elderly individuals often have immune senescence. Decline in immune function may be critical in the disease evolution of HIV-negative PBL.

Since PBL without HIV infection usually has a poorer response to chemotherapy compared with HIV-positive counterparts (2), there is urgent need to develop novel therapies to improve treatment efficacy. Immunotherapy may be promising.

For stage I patients, we first explored the role of radiotherapy. However, the application of radiotherapy did not improve the OS.

In our research, statistical analysis revealed that treatment response had a defined correlation with OS. Either CR or PR was superior to the refractory group in OS (P<0.0001 and P=0.0066, respectively). Furthermore, the CR group showed an OS benefit when compared to PR (P=0.001). Castillo *et al* (54) demonstrated that CR to chemotherapy had increased OS when compared with patients without CR. Differently, our data elucidated that not only CR, but PR also could bring an OS benefit. The different treatment response was derived from tumor heterogeneity. Extensive research on the heterogeneity of HIV-negative PBL may find the causes of treatment resistance and contribute to the improvement of OS.

## Figures and Tables

**Table I:** Clinical features of the HIV-negative PBL cases.

Features	n	%
Age (years) (n=114)		
Mean at diagnosis	58.90	
Range	2–86	
<30	7	6.14
30–60	43	37.72
≥60	64	56.14
Gender (n=114)		
Male	79	69.30
Female	35	30.70
Ann Arbor stage (n=102)		
I	32	31.37
II	18	17.65
III	12	11.76
IV	40	39.22
Bone marrow involvement (n=86)		
With involvement	11	12.79
No involvement	75	87.21
Primary sites (n=114)		
LN	23	20.18
Extra LN	91	79.82
Oral	20	17.54
Nasal cavity and sinus	20	17.54
Gastrointestinal tract	18	15.79
Skin	7	6.14
Other extranodal sites	26	22.81

HIV, human immunodeficiency virus; PBL, plasmablastic lymphoma; LN, lymph node.

**Table II:** Etiological analysis of HIV-negative PBL cases.

Etiology	n	%
EBV infection (n=92)		
With EBV infection	54	58.70
Without EBV infection	38	41.30
Immunosuppression (n=103)		
No immunosuppression	74	71.84
With immunosuppression	29	28.16
Post transplantation	11	10.68
Immune-related disease	8	7.77
Current or previous malignancy	10	9.71
Herpesvirus-8 (HHV-8) (n=53)		
With HHV-8 infection	4	7.55
Without HHV-8 infection	49	92.45

HIV, human immunodeficiency virus; PBL, plasmablastic lymphoma; EBV, Epstein-Barr virus; HHV-8, herpesvirus-8.

**Table III:** Pathological findings of the HIV-negative PBL cases.

IHC analysis	Positive/total tested cases	%
Plasma cell markers		
CD38	22/32	68.75
CD138	67/82	81.71
VS38c	11/11	100.00
MUM1	47/57	82.46
Leukocyte common antigen		
CD45	18/45	40.00
B-cell markers		
CD20	5/87 (4±, 1+)	5.75
CD79a	22/58	37.93
T-cell markers		
CD3	7/47	14.89
CD5	3/23	13.04
NK-cell markers		
CD56	9/42	21.43
Epithelial membrane antigen		
EMA	16/27	59.26
Ki-67	Mean: 83%, n=47	Range: 50–100

HIV, human immunodeficiency virus; PBL, plasmablastic lymphoma; IHC, immunohistochemistry.

**Table IV:** Treatment strategies, response and prognosis of the HIV-negative PBL cases.

Treatment strategy	n	%
Surgery (n=81)		
Received Surgery	15	18.52
No surgery	66	81.48
Radiotherapy (n=90)		
Received radiotherapy	31	34.44
No radiotherapy	59	65.56
Chemotherapy (n=89)		
Received chemotherapy	75	84.27
No chemotherapy	14	15.73
Treatment response (n=71)		
CR	39	54.93
PR	12	16.90
Refractory	18	25.35
Intolerance	2	2.82
Prognosis (n=96)		
Alive	50	52.08
Dead	46	47.92

HIV, human immunodeficiency virus; PBL, plasmablastic lymphoma; CR, complete remission; PR, partial remission.

**Table V:** Univariate analysis of prognostic factors for OS.

	Hazard ratio (95% CI)	P-value
Age (≥60 vs. <60)	1.195 (0.6367–2.243)	0.5792
Gender (M vs. F)	0.8231 (0.4275–1.585)	0.5604
Primary site (oral vs. extraoral)	0.6241 (0.2646–1.472)	0.2816
Ann Arbor stage (I vs. IV)	0.3731 (0.1802–0.7727)	0.0079
Immunosuppression (with vs. without)	0.4114 (0.1920–0.8815)	0.0223
EBV (infection vs. no infection)	2.763 (1.368–5.578)	0.0046
Stage I (with radiotherapy vs. without)	0.7052 (0.1484–3.351)	0.6604
Treatment response (CR vs. refractory)	0.01804 (0.005324–0.06111)	<0.0001
Treatment response (PR vs. refractory)	0.2402 (0.08593–0.6716)	0.0066
Treatment response (CR vs. PR)	0.04391 (0.006841–0.2819)	0.001

OS, overall survival; HIV, human immunodeficiency virus; PBL, plasmablastic lymphoma; EBV, Epstein-Barr virus; CR, complete remission; PR, partial remission.

## Abbreviations

CR: complete remission
EBV: Epstein-Barr virus
HHV-8: herpesvirus-8
HIV: human immunodeficiency virus
OS: overall survival
PBL: plasmablastic lymphoma
PR: partial remission
